# The influence of physiological status on age prediction of *Anopheles arabiensis* using near infra-red spectroscopy

**DOI:** 10.1186/1756-3305-6-298

**Published:** 2013-10-14

**Authors:** Alex J Ntamatungiro, Valeriana S Mayagaya, Stefan Rieben, Sarah J Moore, Floyd E Dowell, Marta F Maia

**Affiliations:** 1 Ifakara Health Institute, Environmental Health and Ecological Sciences, P.O. Box 53, Ifakara, Tanzania; 2 London School of Hygiene & Tropical Medicine, Keppel Street, WC1E 7HT London, UK; 3 Swiss Tropical & Public Health Institute, Soccinstraße 57, 4002 Basel, Switzerland; 4 University of Basel, Petersplatz 1, 4003 Basel, Switzerland; 5 Engineering and Wind Erosion Research Unit, USDA ARS Centre for Grain and Animal Health Research, Manhattan, KS, USA

**Keywords:** *Anopheles arabiensis*, Near infra-red spectroscopy, Age-grading, Physiological status

## Abstract

**Background:**

Determining the age of malaria vectors is essential for evaluating the impact of interventions that reduce the survival of wild mosquito populations and for estimating changes in vectorial capacity. Near infra-red spectroscopy (NIRS) is a simple and non-destructive method that has been used to determine the age and species of *Anopheles gambiae* s.l. by analyzing differences in absorption spectra. The spectra are affected by biochemical changes that occur during the life of a mosquito and could be influenced by senescence and also the life history of the mosquito, i.e., mating, blood feeding and egg-laying events.

**Methods:**

To better understand these changes, we evaluated the influence of mosquito physiological status on NIR energy absorption spectra. Mosquitoes were kept in individual cups to permit record keeping of each individual insect’s life history. Mosquitoes of the same chronological age, but at different physiological stages, were scanned and compared using cross-validations.

**Results:**

We observed a slight trend within some physiological stages that suggest older insects tend to be predicted as being physiologically more mature. It was advantageous to include mosquitoes of different chronological ages and physiological stages in calibrations, as it increases the robustness of the model resulting in better age predictions.

**Conclusions:**

Progression through different physiological statuses of *An. arabiensis* influences the chronological age prediction by the NIRS. Entomologists that wish to use NIR technology to predict the age of field-caught *An. gambiae* s.l from their study area should use a calibration developed from their field strain using mosquitoes of diverse chronological ages and physiological stages to increase the robustness and accuracy of the predictions.

## Background

Mosquito age-grading provides an important source of information on survival rates of vector populations and for understanding malaria epidemiology and transmission [1]. Only older mosquitoes are capable of transmitting the disease because the *Plasmodium* parasites take 8-14 days to develop into the sporozoite stage that is infective to humans [2]. The ability to determine chronological age of the vector provides insight into population structure and vectorial capacity. This is essential for interventions that require an evaluation of their impact on the survival of wild mosquito populations and for the development of life shortening vector control strategies such as slow acting insecticides such as clorophenapyr, new biological control agents like *Wolbachia* and entopathogenic fungi as well attractive toxic sugar baits [3-5]. Alternatively, mark-release recapture technique has been used in a number of investigations to determine the survival and dispersal of mosquitoes transmitting diseases. However, this method is confounded by the low recapture rate which is largely influenced by topographical features, number and type of traps used, weather e.g. wind. Hence, there is a need to capture a large proportion of the population in order to achieve acceptable levels of accuracy [6].

Traditional age grading methods rely on dissection and observation of morphological changes in the mosquito reproductive tract. The most commonly applied age classification technique for mosquitoes is the ovary tracheation method of Detinova [7]. The presence or absence of tracheole skeins is used to classify mosquitoes as either nulliparous or parous, and this proportion is used to calculate the probability of a mosquito surviving one gonotrophic cycle. The changes in the daily survival can be used to calculate changes in mosquito longevity to determine the impact of an intervention on the vectorial capacity of mosquito populations [8]. This dissection method has many limitations as it requires 1) knowledge of the duration of the gonotrophic cycle for that species in that ecological setting, 2) assumes that daily vector survival is constant, although it has been shown to decrease as the mosquito ages [9] 3) assumes that mosquitoes take one blood meal per gonotrophic cycle when some take two before developing the first batch of eggs – the so called pre-gravidae [10]. The Polodova technique is able to identify how many ovipositions a female mosquito has completed by observing the dilatations in the mosquito ovarioles [11]. However, only a handful of entomologists in the world are dexterous enough to perform the Polodova technique. In addition, the results retrieved by these methods are not very accurate, rely on subjective visual observation and are impracticable for large scale studies [12].

Currently, there is a trend in novel age grading methods as researchers search for a simple, scalable assay that can measure vector survival rates in the field. Methods for age prediction by analysis of cuticular hydrocarbons and gene profiling provide accurate chronological age prediction [13,14]; however, the cost for equipment and ongoing analysis limits their routine application. Near-infrared spectroscopy (NIRS) has been previously demonstrated to sufficiently age grade pests of stored cereals [15]. Recently, it has been proven to successfully age-grade and differentiate laboratory reared *An. gambiae* sensu stricto from its morphologically identical sibling *An. arabiensis*[16]. This breakthrough provides a convenient alternative to time-consuming dissection methods and other expensive methods that require high-tech laboratory facilities [17]. NIRS is a non-destructive method that can be used to age grade and speciate thousands of wild mosquitoes in a day [16]. The NIRS method measures energy absorption at specific wavelengths by biological matter, which alters with age of the vector and species. Although not clearly studied, it is thought that compounds found on the cuticular surface of the mosquito, as well as its water composition, determine the absorption spectrum. Differences in water composition even within cryptic species are known to exist, such as with *An. arabiensis* and *An. gambiae* s.s. [18]. Previous studies using NIRS have demonstrated chronological age prediction accuracy >80% for fresh and preserved mosquitoes that were sufficiently old to transmit malaria sporozoites i.e. >7 days old [19]. However, these experiments were performed with mosquitoes that had never blood fed, so their physiological status was not taken into account.

In the present study, we objectively conducted a series of experiments using laboratory-reared *An. arabiensis* of known chronological age and physiological status, to determine how physiological status impacts the NIR energy absorption and thus age prediction. Mosquito physiological status has been proven to influence the composition of cuticular biochemicals [20,21], and it is an important factor for NIRS age determination, as it might change cuticular hydrocarbons and jeopardize the robustness of the tool.

## Methods

### Laboratory reared mosquitoes

*An. arabiensis* were reared inside a screened house semi-field system under ambient conditions [22]. Approximately 1000-1500 eggs from the adults’ cage were put into the 250 ml hatching bowl lined with the wetted filter paper. Around 150 ml of water was added into the hatching bowl and then left under natural sunlight in the screened house. Following hatching, larvae were added to 3.5 L water in a basin. Approximately 0.5 g of Tetramin fish food was then added on a daily basis, and the larvae were reared under natural photoperiod 12:12 Light: Dark. After 8-10 days depending on the weather conditions developing pupae were collected into a 250 ml bowl that was then put into a prepared cage for emergence. Adult females were blood-fed by inserting a human (volunteer) arm inside the cage each time for 15 minutes (Ethical clearance No. IHRDC/EC4/CL.N96/2004) and provided with fresh 10% glucose solution daily. Blood feeding was always carried out after a 6-hour starvation period where 10% glucose source was withdrawn to ensure avidity.

### Mosquito handling

Upon emergence for each replicate, 50 female mosquitoes were released in a netted cage (40×40 cm) together with 150 male mosquitoes (3-days or older). A 3 to 1 proportion was chosen to increase the chances of mating occurring. On the third day post-emergence mosquitoes were offered a blood meal. The following morning, all females were carefully aspirated using a siphon and transferred into individual styrofoam cups. The bottom of the cups was lined with moistened filter paper to allow oviposition. After laying an egg-batch, mosquitoes were transferred into new cups. Blood meals were offered *ad libitum* every day; around 19.00 hrs volunteers placed their forearm over the cups and allowed mosquitoes to feed through the netting material for a period of 15 minutes.

Details of each mosquito’s life history were recorded individually, by documenting when each mosquito blood-fed and laid eggs. Shortly before NIR scanning, mosquitoes were killed using chloroform vapour in a well-ventilated room, after scanning they were submitted to dissection of the spermathecae to confirm if mating had occurred. Briefly, a drop of water was added on a clean slide, the thorax was gently grasped using a pair of forceps and placed ventral side up with the abdomen resting in the water under the stereoscope. Gently the terminalia was removed by grasping them and pulling away slowly using a fine tip needle or forceps. The spermatheca was relocated within the 8^th^ segment and terminalia section removed previously. A cover slip was gently lowered onto the spermatheca using a needle (to avoid rupturing the spermathecae). On the underside of the slide, the area surrounding the spermathecae was circled using a permanent marker. A compound microscope magnification with 100X was used to observe movement of the long thread-like spermatozoa within the spermathecae [23]. Mosquitoes were considered mated, when sperm were clearly observed within the spermathecae. Unclean or uncertain samples were excluded from the analysis.

### Experimental design

Mosquitoes were scanned on four fixed chronological ages, i.e. 3, 5, 8 and 11 days. This allowed us to compare differences in spectra between mosquitoes of the same age but in different physiological stages (virgin, pre-gravid, 1^st^ oviposition, 2^nd^ oviposition). A minimum of 25 mosquitoes were scanned for each combination of age and physiological age. The scanning protocol has previously been described elsewhere [11].

### Data analysis

ASD software RS^3^ (Version 3.1) was used to collect all spectra. These were converted to GRAMS format (Thermo Galactic, Salem, NH, USA) using ASD ViewSpecPro. The Grams software PLSPlus/IQ was used to perform partial least squares (PLS) regression analysis on the spectra for cross-validation as described previously [16]. Spectra of *An. arabiensis* were collected at the chronological ages of 3, 5, 8, and 11 days, and physiological statuses of virgin, pre-gravid, 1^st^ oviposition, and 2^nd^ oviposition (assigned 1, 2, 3, 4 for PLS regression analysis). To determine the effect of physiological status in a particular age group, the average of predicted values for each physiological status were compared. To quantify the effects of both actual chronological age and physiological status on the age predictions, a generalized linear model (GLM) fitted to a Gaussian distribution was performed using prediction value as the dependant variable and the physiological age as the independent value for each actual category.

## Results and discussion

### Chronological age predictions

**Figure 1:**
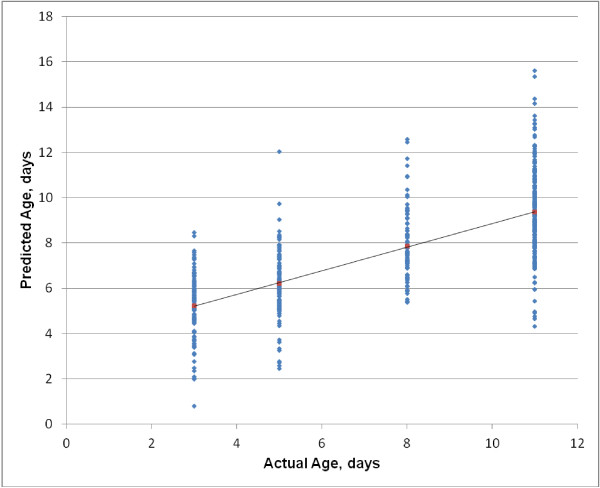
**NIRS predicted chronological age versus the actual age of *An. arabiensis.*** Chronological age predictions by NIRS using model that includes all physiological stages (virgin, pre-gravid, 1^st^ oviposition, 2^nd^ oviposition).

The objective of this study was to determine if the progression through different physiological changes would alter the NIRS chronological age prediction of *An. arabiensis*. In order to investigate models to predict chronological age, we first developed a calibration using pre-gravid mosquitoes of different chronological age to predict the age of other mosquitoes at other physiological stages. Results showed that the predicted chronological age increased along with the real age (Figure 1), but younger mosquitoes were predicted as older than their actual age, and older mosquitoes were predicted as younger than their actual age.

However, predictions generated from a cross validation that included mosquitoes of different physiological stages neared the actual chronological age, meaning that age prediction accuracy was increased (Table 1). Therefore, it seems advantageous to include mosquitoes of different physiological stages in calibrations, as it increases the robustness of the model by introducing sources of variability, proving the importance of including mosquitoes of all physiological stages when developing a calibration to predict field samples. Robustness of a calibration increases as the physiological diversity of mosquitoes in each age class increases. Therefore, a calibration for NIRS age prediction of any wild mosquito population should include mosquitoes of different stages that have mated, blood fed and laid eggs in order to represent the diversity of mosquitoes through the natural stages of their lifetime [24].

**Table 1:** **The mean and standard error of chronological age predictions using NIRS of female *An. arabiensis***

**Predictions from pre-gravidae calibration**	**Pre-gravid**		**Virgin**		**1**^**st **^**oviposition**		**2**^**nd **^**oviposition**		
	**n**	**Prediction**	**n**	**Prediction**	**n**	**Prediction**	**n**	**Prediction**	**p-values**
**Chronological age**									
**3 days-old**	58	**NA**	43	(4.4) ±1.65	-	-	-	-	-
**5 days-old**	43	**NA**	39	(5.5) ± 1.25	38	(5.6) ±1.44	-	-	0.641
**8 days-old**	38	**NA**	26	(7.6) ± 1.54	47	(6.4) ± 0.95	-	-	0.014
**11 days-old**	48	**NA**	42	(8.4) ± 1.78	68	(7.9) ± 2.12	69	(8.6) ±1.61	0.592
**Predictions from a cross-validation**	**Pre-gravid**		**Virgin**		**1**^**st **^**oviposition**		**2**^**nd **^**oviposition**		
	**n**	**Prediction**	**n**	**Prediction**	**n**	**Prediction**	**n**	**Prediction**	**p-values**
**Chronological age**									
**3 days-old**	58	(3.7) ± 1.24	43	(4.0) ± 1.23	-	-	-	-	0.730
**5 days-old**	43	(5.2) ± 1.77	39	(6.1) ± 2.59	38	(5.9) ± 1.42	-	-	0.225
**8 days-old**	38	(7.9) ± 1.68	26	(6.8) ± 2.14	47	(8.0) ± 1.66	-	-	0.850
**11 days-old**	48	(10.2) ± 2.12	42	(10.4) ± 1.94	68	(10.6) ± 2.62	69	(9.91) ± 2.90	0.099

The p-values were generated from a generalized linear model fitted to a Gaussian distribution analyzing differences between age predictions of mosquitoes at different physiological stages but within the same age group.

### Physiological status prediction

We investigated the robustness of NIRS to determine if it is capable of predicting the physiological age of mosquitoes (prediction values were: virgin=1.0; pre-gravid=2.0; 1^st^ oviposition=3.0; 2^nd^ oviposition=4.0). We observed a slight trend within some physiological stages of the same chronological age group suggesting that older insects tend to be predicted as being physiologically more mature. For example, 3-day old virgin mosquitoes had their physiological status correctly predicted as virgin (prediction value 1.08), but 11-day old mosquitoes were predicted as pre-gravidae (prediction value 2.44) (Table 2). Ideally virgin mosquitoes should have a predicted physiological status of 1.0. Similar trends were also seen for pre-gravid and mosquito that laid one egg batch.

**Table 2:** **The mean predicted physiological age of *An. arabienis *using NIRS**

**Chronological age**	**Virgin**	**Pre-gravid**	**1**^**st **^**oviposition**	**2**^**nd **^**oviposition**
3 days old	1.08 ± 0.49	1.98 ± 0.28		
5 days old	1.87 ± 0.46	2.05 ± 0.44	2.19 ± 0.39	
8 days old	2.03 ± 0.40	2.13 ± 0.48	2.33 ± 0.30	
11 days old	2.44 ± 0.34	2.5 ± 0.23	2.62 ± 0.45	2.6 ± 0.32
**Average**	**2.085 ± 0.42**	**2.165 ± 0.36**	**2.38 ± 0.38**	**2.6 ± 0.32**

The mean physiological ages were predicted from the cross-validation model developed using *An. Arabiensis* of different physiological status (Virgin-1.0, Pre-gravid=2.0, 1^st^ oviposition=3.0, 2^nd^ oviposition=4.0).

The effects of physiological status on the age predictions, the age prediction values through different physiological statuses in each actual age category were not significantly different (Table 1). Thus, NIRS could not tell the difference between a virgin mosquito, pre-gravid mosquito, a mosquito that laid one egg batch or a mosquito that laid two egg batches. Prediction values from progressive physiological status (virgin to pre-gravid to 1^st^ oviposition and to 2^nd^ oviposition) were not significantly different; hence the NIRS cannot be used to determine physiological age. However, there were trends in the predicted values along the different physiological stage, this may imply that changes in organic constituents in multi-parous mosquitoes occur but are not substantial enough to affect near infra red energy absorption spectra. We observed a decreased accuracy of age prediction in older mosquitoes as seen in the broad range in predicted age of the 11 day old insects, consistent with previous studies using laboratory and semi field reared mosquitoes [16], this may be due to a decrease in aging associated biochemical changes in the thoracic cuticle [25,26]. Similarly, mosquito cuticular water contents have been implicated with the characteristic NIR energy absorption spectra [18,26,27]. Though, according to our observation the changes in physiological status of *An. arabiensis* probably do not affect their water content.

## Conclusions

A study using gas chromatography-mass spectrometry to characterize the epicuticular surface of the *An. gambiae* s.s, demonstrated the association of quantitative relative abundance of cuticular C-H functional groups with physiological changes of the mosquito [20]. This observation may give us clues on how physiological changes along the lifetime of *An. arabiensis* are reflected biochemically and therewith affect NIR absorption spectra. Perhaps, the NIR age grading is formed on the basis of spectra difference corresponding to C-H and C=O important overtones likely caused by absorption by cuticular lipids and water respectively [25,28]. Further research is necessary to better understand the factors that influence changes in NIR absorption of *An. arabiensis* mosquitoes along their lifetime. We now know that progression through both chronological and physiological age have an influence on the absorption spectra although not substantial enough to allow the prediction of physiological age. Entomologists that wish to use the NIR technology to predict the age of field-caught *An. gambiae* s.l. from their study area should use a calibration performed from their field strain using mosquitoes of diverse chronological ages as well as diverse physiological stages in order to increase the robustness and accuracy of the predictions. Our finding demonstrates that physiological status of *An. arabiensis* is an important factor in a model for chronological age grading. Robustness of the calibration model improves as the physiological diversity of mosquitoes in each age class increases. Further analysis of insect cuticle may reveal the biochemistry underlying the changes seen from NIR spectra, which could potentially be applied to improve the prediction of chronological age and physiological status of malaria vectors.

## Competing interests

All authors declare that they have no competing interests.

## Authors’ contributions

AJN drafted the manuscript; SJM, MFM conceived the study; AJN, SJM and MFM designed the experiments; AJN and SR ran the experiments; FED and VSM analysed data; SR, VSM, SJM, MFM and FED reviewed the manuscript. All authors read and approved the final manuscript.
